# A Comparative Analysis of the Efficacy and Safety of Hot Snare Polypectomy and Cold Snare Polypectomy for Removing Small Colorectal Polyps: A Systematic Review and Meta-Analysis

**DOI:** 10.7759/cureus.38713

**Published:** 2023-05-08

**Authors:** Kevin Winston, Hasan Maulahela, Daniell Edward Raharjo, Kevin Tjoa, Reganedgary Jonlean

**Affiliations:** 1 Hospital Medicine, Bhakti Medicare Hospital, Sukabumi, IDN; 2 Gastroenterology, Cipto Mangunkusumo National General Hospital, Jakarta, IDN; 3 Faculty of Medicine, Universitas Indonesia, Jakarta, IDN; 4 Stem Cell Transplantation Unit, Tzu Chi Hospital, Jakarta, IDN

**Keywords:** colorectal polyp, polypectomy, bleeding, complete resection, gastroenterology

## Abstract

Both cold snare polypectomy (CSP) and hot snare polypectomy (HSP) have been shown to be effective methods for removing small colorectal polyps, but the optimal method for achieving complete resection remains unclear. To address this issue, we conducted a systematic search of relevant articles using databases such as PubMed, ProQuest, and EBSCOhost. The search criteria included randomized controlled trials that compared CSP and HSP for small colorectal polyps ≤10 mm and the articles were screened based on specific inclusion and exclusion criteria. The data were analyzed using RevMan software (version 5.4; Cochrane Collaboration, London, United Kingdom), and meta-analysis was performed with outcomes measured using pooled odds ratios (OR) and 95% confidence intervals (CI). The Mantel-Haenszel random effect model was used to calculate the OR. We selected a total of 14 randomized controlled trials involving 11601 polyps for analysis. Pooled analysis showed no statistically significant difference in the incomplete resection rate between CSP and HSP (OR: 1.22; 95% CI: 0.88-1.73, p-value: 0.27; I^2^: 51%), en bloc resection rate (OR: 0.66; 95%CI: 0.38-1.13; p: 0.13; I^2^: 60%), and polyp retrieval rate (OR: 0.97; 95%CI: 0.59-1.57; p: 0.89; I^2^: 17%). For safety endpoints, there is no statistically significant difference in intraprocedural bleeding rate between CSP and HSP per patient analysis (OR: 2.37, 95% CI: 0.74-7.54; p: 0.95; I^2^: 74%) and per polyp basis (OR: 1.84, 95% CI: 0.72-4.72; p: 0.20; I^2^: 85%). CSP had lower OR for the delayed bleeding outcome when compared with the HSP group per patient basis (OR: 0.42; 95% CI: 0.2-0.86; p: 0.02; I^2^: 25%), but not in the per polyp analysis (OR: 0.59; 95% CI: 0.12-3; p: 0.53; I^2^: 0%). Total polypectomy time was significantly shorter in the CSP group (mean difference: -0.81 minutes; 95% CI: -0.96, -0.66; p:<0.00001; I^2^: 0%). Thus, CSP is both an efficacious and safe method for removing small colorectal polyps. Therefore, it can be recommended as a suitable alternative to HSP for the removal of small colorectal polyps. However, more studies are necessary to evaluate any long-term differences between the two methods such as polyp recurrence rates.

## Introduction and background

According to data from the Global Cancer Observatory (GLOBOCAN) in 2020, colorectal cancer (CRC) is the third most common cancer in the world and the second leading cause of cancer deaths [1]. Therefore, it is of utmost importance to conduct regular screening for colorectal cancer and to provide timely treatment, particularly in the early stages when most CRC begins as polyps. Many studies have demonstrated that screening colonoscopy with polyp removal can significantly reduce mortality from colorectal cancer [2,3]. From an epidemiological perspective, this approach is crucial to decrease the burden of CRC and improve overall public health.

Snare polypectomy with electrocautery, or hot snare polypectomy (HSP), is a widely used technique for removing colorectal polyps sized ≤10 mm. The use of electrocautery during HSP is thought to decrease intraprocedural bleeding and eliminate neoplastic cells at the tissue margin area [4]. However, these potential benefits may be accompanied by an increased risk of delayed bleeding. For example, a retrospective study by Chang et al., with 2529 patients, observed that CSP produced a lower rate of delayed bleeding [5]. Moreover, HSP has been associated with postpolypectomy electrocoagulation syndrome, which can cause symptoms resembling colon perforation due to the electric current extending beyond the mucosal tissue [6,7]. As a result, many gastroenterologists are turning to polypectomy without electrocautery, known as cold snare polypectomy (CSP), as a safer alternative to HSP [5,8].

Despite its benefits, CSP also has some disadvantages. One of the major concerns is its relatively low complete resection rate, which can increase the risk of polyp recurrence and have negative effects on patients [9]. Additionally, there is a lack of evidence on which polypectomy technique is most appropriate for small polyps, highlighting the need for further research. To address this gap, we conducted a systematic review and meta-analysis to compare the effectiveness and safety of HSP and CSP in removing colorectal polyps sized ≤10 mm.

## Review

This review has been registered with PROSPERO (International Prospective Register of Systematic Reviews) by the National Institute for Health Research (NIHR) with ID CRD42021227043.

Search strategy

The authors conducted a systematic literature search on three medical databases (PubMed, ProQuest, and EBSCOhost) from February 2022 to February 2023. The keywords and their synonyms used for the systematic search were: colon polyp, colorectal cancer, cold snare polypectomy, hot snare polypectomy, complete resection rate, and bleeding. The search was restricted to articles in English and Indonesian. All full-text articles were obtained using institutional access at Universitas Indonesia and American College of Physician membership.

Inclusion and Exclusion Criteria

We conducted a systematic review and meta-analysis of randomized controlled trials published within the last 15 years that compared cold snare polypectomy with hot snare polypectomy. Our inclusion criteria were: (1) adult patients aged 18 years or older; (2) colorectal polyps sized 10 mm or smaller; (3) outcome of complete resection rate or incomplete resection rate; (4) outcome of en bloc resection; (5) outcome of polyp retrieval time; (6) outcome of procedural bleeding or delayed bleeding and/or bleeding; and (7) study design of randomized controlled trials. We excluded observational studies, poster presentations, studies not in English or Indonesian, and animal studies.

Study Selection

The initial screening of articles was based on their titles and abstracts. Duplicates were manually cross-checked, and any duplicated articles were removed from the search results. Subsequently, independent full-text screening was conducted on the remaining articles. All authors independently examined the articles, and any disagreements were resolved through consensus.

Data Extraction and Outcome

The following information was extracted from selected articles: name of the first author, year of publication, study location, sample size, and demographical data. The measured outcomes are complete resection rate, early bleeding rate, delayed bleeding rate, perforation, and procedural time. All information was verified by two authors.

Statistical Analysis

All statistical analyses were conducted using RevMan software (version 5.4; Cochrane Collaboration, London, United Kingdom). Outcomes were measured with pooled odd ratios (OR) with 95% confidence intervals (CI). OR was calculated under the Mantel‐Haenszel random effect model. Meanwhile, the heterogeneity of the pooled analysis was measured by the chi‐squared test and I^2^ statistic. Low, moderate, and high heterogeneity were associated with I^2^ values of 25%, 50%, and 75% with an I^2^ value of higher than 50% considered as having significant heterogeneity [10]. P < 0.05 was considered statistically significant.

Result

**Figure 1:**
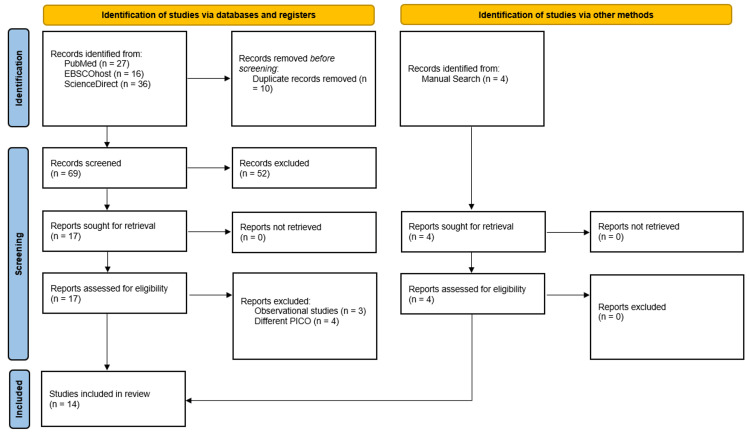
PRISMA Flowchart PRISMA: Preferred Reporting Items for Systematic Reviews and Meta-Analyses

Figure 1 shows the Preferred Reporting Items for Systematic Reviews and Meta-Analyses (PRISMA) flowchart of the literature selection process. A systematic search of multiple medical databases resulted in 79 unique studies, of which 10 duplicates were removed. After screening the titles and abstracts of the remaining 69 studies, 17 articles were selected for full-text reading. Seven of these were subsequently excluded, leaving 10 articles that met the criteria for inclusion in the systematic review and meta-analysis. Additionally, a manual search yielded four more articles that met the inclusion criteria, bringing the total number of articles used in this study to 14 (Table 1) [11-24].

**Table 1:** Characteristics of the 14 studies used for systematic review and meta‐analysis

Author	Year	Country	Study Design	Randomization Method	Sample Size	Patient	Intervention	Comparison	Outcome
Chang et al [11]	2023	Taiwan	Multicenter RCT	Computer-generated random sequence	4270 patients	4-10 mm polyps with concurrent polyps larger than 10 mm (Data on patients with polyps ≤10 mm is available on supplement)	Cold snare polypectomy	Hot snare polypectomy	Delayed bleeding, severe bleeding, mean polypectomy time, successful tissue retrieval, en bloc resection, complete histologic resection, emergency service visits
Pedersen et al [12]	2022	Norway, Poland, Denmark, and USA	Multicenter RCT	Block randomization	425 patients	4-9 mm non-pedunculated polyps	Cold snare polypectomy	Hot snare polypectomy	Complete resection rate, early/immediate bleeding, delayed bleeding
Sanz et al [13]	2020	Spain	RCT	Computer-generated random sequence	496 patients	5-9 mm polyps	Cold snare polypectomy	Hot snare polypectomy	Complete polypectomy rates, intraprocedural bleeding, delayed bleeding, post-colonoscopy abdominal pain
Ito et al [14]	2021	Japan	RCT	Numbered container method	119 patients	6-9 mm polyps	Cold snare polypectomy	Hot snare polypectomy	Delayed bleeding Immediate bleeding, complete resection, en bloc resection, perforation withdrawal time
Aizawa et al [15]	2019	Japan	RCT	Computer-generated random sequence	273 patients	≤ 9 mm colorectal polyps	Cold snare polypectomy	Hot snare polypectomy	Delayed bleeding rates, immediate bleeding rates, clipping rates, early bleeding
Takeuchi et al [16]	2019	Japan	RCT	Computer-generated random sequence	184 patients	Subcentimeter colorectal polyps	Continuous anticoagulation + cold snare polypectomy	Heparin bridging + hot snare polypectomy	Polypectomy-related major bleeding, mean procedure time, mean hospital stay, adverse events
Papastergiou et al [17]	2018	Greece	RCT	Block randomization	155 patients	6-10 mm nonpedunculated colorectal polyps	Cold snare endoscopic mucosal resection	Hot snare endoscopic mucosal resection	Histological complete resection, intraprocedural bleeding, postprocedural bleeding, or perforation
Suzuki et al [18]	2017	Japan	RCT	Block randomization	52 patients	Rectal or rectosigmoid polyps ≤10 mm	Cold snare polypectomy	Hot snare polypectomy	Mucosal defect diameter, en bloc resection, complete resection, perforation, delayed bleeding
Zhang et al [19]	2017	Japan	RCT	Sealed envelope system	358 patients	Polyp size 6-9 mm	Cold snare polypectomy	Hot snare polypectomy	Incomplete resection rate, total procedural time, procedural bleeding
Kawamura et al [20]	2018	Japan	RCT	Block randomization	538 patients	4–9 mm colorectal sessile adenomatous polyps	Cold snare polypectomy	Hot snare polypectomy	Complete resection rate, postoperative bleeding, polyp retrieval rate, procedure time
Gomez et al [21]	2015	USA	RCT	Not mentioned	60 patients	Diminutive polypectomy (< 6 mm)	Cold snare polypectomy	Hot snare polypectomy	Adequacy of resection of diminutive polyps
Horiuchi et al [22]	2015	Japan	RCT	Block randomization	70 patients	Colorectal polyps up to 10 mm	Cold snare polypectomy	Hot snare polypectomy	Immediate bleeding delayed bleeding Complete polyp retrieval rates, presence of histologically demonstrated injured arteries
Paspatis et al [23]	2011	Greece	RCT	Bernoulli process	414 patients	Polyps 3-8 mm	Cold snare polypectomy	Cot snare polypectomy	early or late postpolypectomy bleeding, Intraprocedural bleeding
Ichise et al [24]	2011	Japan	RCT	Sealed envelope system	80 patients	Colorectal polyps up to 8 mm	Cold snare polypectomy	Cot snare polypectomy	Procedure time, complete polyp retrieval rates, bleeding requiring hemostasis, abdominal symptoms

The oldest article included in this analysis was published in 2011 by Ichise et al. while the most recent one was by Chang et al. in 2023 [11,24]. Out of the 14 studies analyzed, eight were conducted in Japan, two in Greece, one in Taiwan, one in the United States, one in Spain, and one in multiple countries. The study by Gomez et al. was the only one that did not specify the method of randomization used [21]. The sample sizes of the studies ranged from 52 to 4270 patients. Notably, the studies by Horiuchi et al., Takeuchi et al., and Chang et al. included a significant patient population that used anticoagulation [11,16,22].

Incomplete Resection Rate

**Figure 2:**
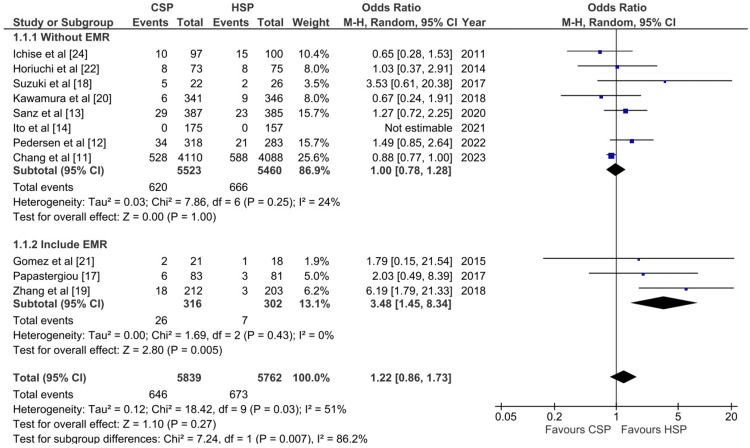
Forest plot of CSP versus HSP with the outcome of incomplete resection rate CSP: cold snare polypectomy; HSP: hot snare polypectomy [11-14,17,19-22,24]

Based on a pooled analysis of 11601 polyps, there was no statistically significant difference between CSP and HSP in terms of the incomplete resection rate outcome (OR: 1.22; 95% CI: 0.88-1.73, p-value: 0.27; I^2^: 51%) (Figure 2). However, due to significant heterogeneity among the studies, we conducted a subgroup analysis. We divided the studies into two categories: those that include the endoscopic mucosal resection (EMR) technique and those without. Interestingly, the pooled studies of studies that included EMR showed that CSP had a higher OR of incomplete resection when compared with HSP (OR: 3.48; 95% CI:1.45-8.34, p-value: 0.005; I^2^: 0%). The heterogeneity in the pooled analysis of studies that did not use EMR was 24% and those that did use EMR was 0%, indicating that the use of EMR is a major cause of the heterogeneity. During EMR, a solution and dye are injected into the submucosa, causing tissue elevation that theoretically facilitates easier access to the area being operated on, which may improve outcomes such as the complete resection rate.

To reliably confirm the complete resection of the polyp, it is recommended to use biopsy/histological assessment. All studies used a biopsy/histological assessment except for the studies by Horiuchi et al. and Ichise et al., which did not mention the method of assessment [22,24].

En Bloc Resection Outcome

**Figure 3:**
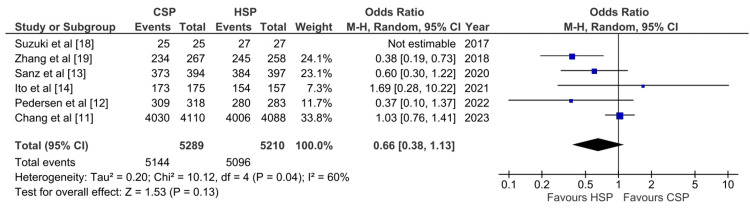
Forest plot of CSP versus HSP with the outcome of en bloc resection [11-14,18,19] CSP: cold snare polypectomy; HSP: hot snare polypectomy

A total of six studies evaluated the en bloc resection outcome. From the pooled analysis, there is no significant difference between CSP and HSP (OR: 0.66; 95%CI: 0.38-1.13; p: 0.13; I^2^: 60%) (Figure 3). The study by Suzuki et al. achieved 100% en bloc resection in both the CSP and HSP groups, thus could not be analyzed in the pooled analysis. Excluding the study by Zhang et al. that used EMR only for the HSP group decreases heterogeneity from 60% to 29% (Appendix 1).

Polyp Retrieval Rate

**Figure 4:**
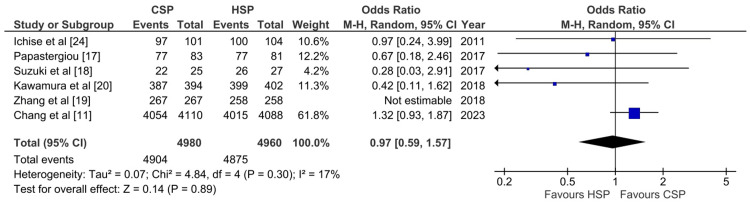
Forest plot of CSP versus HSP with the outcome of polyp retrieval rate [11,17-20,24] CSP: cold snare polypectomy; HSP: hot snare polypectomy

Only five studies were able to be analyzed for the pooled analysis. The result shows that no statistically significant difference in polyp retrieval rate between CSP and HSP is detected (OR: 0.97; 95%CI: 0.59-1.57; p: 0.89; I2: 17%) (Figure 4).

Intraprocedural Bleeding Outcome

**Figure 5:**
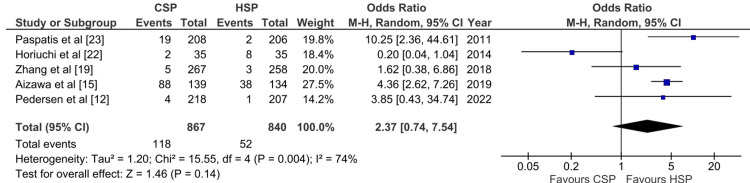
Forest plot for meta‐analysis of CSP versus HSP with the outcome of delayed bleeding rate on a per-patient basis [12,15,19,22,23] CSP: cold snare polypectomy; HSP: hot snare polypectomy

**Figure 6:**
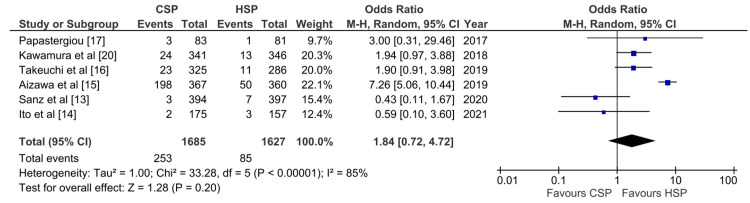
Forest plot of CSP versus HSP with the outcome of intraprocedural bleeding per polyp [13-17,20] CSP: cold snare polypectomy; HSP: hot snare polypectomy

Five studies reported intraprocedural outcomes on a per-patient basis and six studies reported on a per-polyp basis. In the pooled analysis, there was no statistically significant difference in intraprocedural bleeding rate between CSP and HSP on a per-patient basis (OR: 2.37, 95% CI: 0.74-7.54; p: 0.95; I^2^: 74%) and per-polyp basis (OR: 1.84, 95% CI: 0.72-4.72; p: 0.20; I^2^: 85%) (Figures 5, 6). Excluding the study conducted by Horiuchi et al. decreases heterogeneity significantly from 74% to 5% (Appendix 2).

Delayed Bleeding Outcome

**Figure 7:**
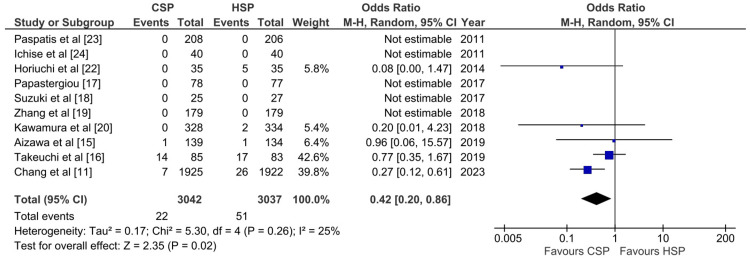
Forest plot of CSP versus HSP with the outcome of delayed bleeding per patient [11,15-20,22-24] CSP: cold snare polypectomy; HSP: hot snare polypectomy

**Figure 8:**
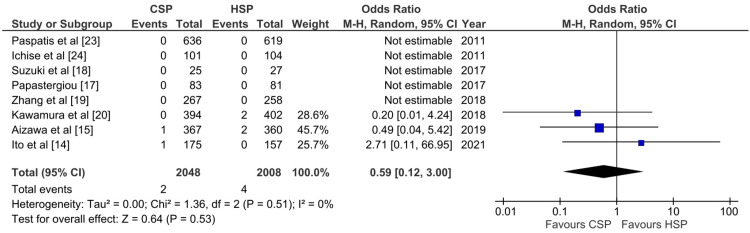
Forest plot of CSP versus HSP with the outcome of delayed bleeding per polyp [14,15,17-20,23,24] CSP: cold snare polypectomy; HSP: hot snare polypectomy

Ten studies compared CSP and HSP in terms of delayed bleeding outcome per patient, but only eight studies provided data on delayed bleeding outcome per polyp. A total of 6079 patients were available for pooled analysis (Figure 7). Results showed that the CSP group had a lower OR for the delayed bleeding outcome when compared with the HSP group on a per-patient basis (OR: 0.42; 95% CI: 0.2-0.86; p: 0.02; I^2^: 25%). However, five studies did not report any cases of delayed bleeding in either group, so no analysis could be conducted for these studies. Overall, the pooled analysis did not reveal significant heterogeneity between the studies. In contrast, the pooled analysis for delayed bleeding per polyp showed no significant difference between CSP and HSP (OR: 0.59; 95% CI: 0.12-3; p: 0.53; I^2^: 0%) (Figure 8). A possible explanation includes a lack of a delayed bleeding event for the per polyp analysis.

Polypectomy Time

**Figure 9:**
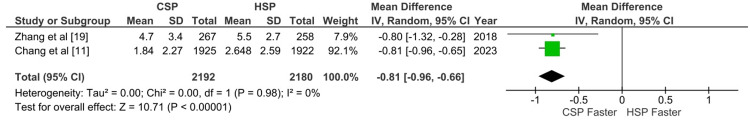
Forest plot of CSP versus HSP with the outcome of procedural time [11,19] CSP: cold snare polypectomy; HSP: hot snare polypectomy

Two studies evaluated polypectomy time. The pooled analysis of the studies, which included 4372 polyps, showed that CSP was significantly faster than HSP (mean difference: -0.81 minutes; 95% CI: -0.96, -0.66; p:<0.00001; I2: 0%) (Figure 9). Thus, on average, using CSP for a polyp is faster by approximately 0.81 minutes (48.6 seconds). The study by Kawamura et al. also measured polypectomy time but used median instead of mean, so it could not be included in the pooled analysis. Nevertheless, the study by Kawamura et al. also found that CSP was faster than HSP (60 seconds versus 83 seconds; p: <0.001).

Discussion****

Colorectal cancer is one of the most common types of cancer in the United States, and it is the second leading cause of cancer deaths [1]. It is more common in people over the age of 50, but it can also affect younger people [25]. Risk factors for colorectal cancer include a family history of the disease, a diet high in red meat and processed foods, inflammatory bowel diseases, such as Crohn's disease and ulcerative colitis, and a sedentary lifestyle [26].

Colorectal cancer usually evolves from polyps. Most polyps are benign, but some polyps have the potential to become cancerous over time. Thus, screening and removal of polyps are important to prevent progression to colorectal cancer.

The recommended age for colon cancer screening varies depending on the guidelines and the person's risk factors. Both the American Cancer Society (ACS) and the United States Preventive Services Task Force (USPSTF) recommend that most adults at average risk of colorectal cancer should begin regular screening at age 45 [27,28]. Once colon polyps are found, they are usually biopsied and/or removed.

Currently, endoscopic techniques for the removal of small colorectal polyps involve cold forceps polypectomy and snare polypectomy. Biopsy forceps are commonly used only for diminutive polyps as their complete resection rate is lower for small polyps. The use of cold forceps polypectomy for diminutive polyps is currently controversial. A study by Lee et al. showed that even for diminutive polyps, cold snare polypectomy is superior to cold forceps polypectomy in achieving a complete resection rate [29]. In contrast, a new study by Wei et al. published in 2022 using 179 patients showed noninferiority of cold forceps to CSP in terms of complete resection rate for nonpedunculated polyps ≤3 mm [30].

According to the European Society for Gastrointestinal Endoscopy's 2017 guideline, cold snare polypectomy is the preferred method for removing diminutive polyps (those that are less than 5 mm in size) [8]. For polyps that are 6-9 mm in size, cold snare polypectomy should be considered due to its safety profile, but the guidelines note that there is currently a lack of evidence on the efficacy of cold snare polypectomy compared to hot snare polypectomy [8]. For polyps sized 10 - 19 mm, European guidelines recommend HSP instead of CSP. In contrast, the US Multi-Society Task Force on Colorectal Cancer recommends either CSP or HSP for non-pedunculated polyps sized 10-19 mm [31].

It is widely accepted that colonoscopy and removal of colorectal polyps is an integral part of CRC incidence and mortality reduction. However, it should be noted that the benefits of the removal of colorectal polyps are influenced by adequate detection and complete resection rate [32]. If either of these factors is lacking, the benefit of colonoscopy will be reduced. For example, the incomplete resection rate is associated with up to 30% of post-colonoscopy cancers [32]. Another study showed that a low adenoma detection rate is associated with a risk of subsequent colorectal cancer and mortality [33]. This means that the complete resection rate is a very important marker for patients, but currently, it is undetermined which polypectomy technique is superior for small polyps.

Our systematic review and meta-analysis consisted of 14 RCT studies. Out of these, 11 studies were used for the incomplete resection rate outcome pooled analysis. The study with the highest sample size was a multicenter randomized trial conducted by Chang et al. [11]. This study observed no statistically significant differences between CSP and HSP for incomplete resection rate [11]. The second largest study conducted by Sanz et al. also showed no differences between CSP and HSP [13]. Only the study by Zhang et al. demonstrated that CSP had a higher incomplete resection rate than HSP [13]. This discrepancy may be explained through the use of HSP together with EMR in the study by Zhang et al. while the CSP group did not use EMR, which may reduce the incomplete resection rate in the HSP group [13]. Thus, from the current studies, it can be concluded that CSP has a similar incomplete resection rate to HSP.

En-bloc resection is an important outcome that endoscopists should strive for, as literature has shown that en-bloc reduces the recurrence of malignancy [34,35]. Thus, both the incomplete resection rate and the en bloc resection rate may be indirectly related to the patient’s survival. Our meta-analysis revealed no difference in the rate of en bloc resection or incomplete resection between CSP and HSP, suggesting that there is no difference in effectiveness between the two methods.

Whether the outcome of the polyp retrieval rate is important is still debatable, as there may not be a need for histological analysis of resected polyps [36]. According to a study by Fernandes et al., the factors associated with polyp retrieval failure include previous colorectal surgery, cold snare polypectomy, right-side location of polyps, inadequate bowel preparation, and polyps ≤5 mm [36]. In contrast, this meta-analysis showed no significant difference between cold and hot snare in the polyp retrieval rate detected (OR: 0.97; 95%CI: 0.59-1.57; p: 0.89) with no heterogeneity (I^2^: 0%). However, it should be noted that only five studies were able to be used for meta-analysis. Future studies may be needed to confirm whether cold snare polypectomy produces a lower polyp retrieval rate.

The bleeding rate, which includes immediate bleeding during the procedure or delayed bleeding after the procedure, is an important outcome to consider. It is widely believed that HSP can cause worse bleeding and long-term bleeding due to tissue damage from the temperature [5,37]. Despite this issue, HSP may still be preferred due to the benefit of cancer cell destruction from the temperature [38]. The results of this meta-analysis indicate that there are no statistically significant differences in intraprocedural bleeding between CSP and HSP. However, for the outcome of delayed bleeding, the per-patient analysis showed that CSP had a lower odds ratio for delayed bleeding but not in the per-polyp analysis.

There are several limitations in our meta-analysis. First, there was a lack of a follow-up colonoscopy in all studies. This means that there is no further confirmation of the complete resection rate of the patients. Second, it is impossible to conduct blinding in all studies due to the study design. Therefore, there may be a potential for bias affecting the complete resection rate. Furthermore, as endoscopy is a skill-based procedure, there may be differences in skills used by endoscopists that could affect the bias of the meta-analysis as more experienced endoscopists have a higher complete resection rate [39]. The differences in experience are very difficult to analyze in these studies.

## Conclusions

A pooled analysis of 14 studies has demonstrated that cold snare polypectomy (CSP) is a safe and effective alternative to hot snare polypectomy (HSP) for the removal of small colorectal polyps (≤10 mm). The analysis revealed that CSP is non-inferior to HSP in terms of incomplete resection rate, en bloc resection, polyp retrieval rate, and procedural bleeding. For delayed bleeding, CSP reduces the risk of delayed bleeding only when analyzed on a per-patient basis. Thus, CSP is both an efficacious and safe method for removing small colorectal polyps. Therefore, it can be recommended as a suitable alternative to HSP for the removal of small colorectal polyps. However, more studies are necessary to evaluate any long-term differences between the two methods such as polyp recurrence rates.

## Figures and Tables

**Figure 10:**
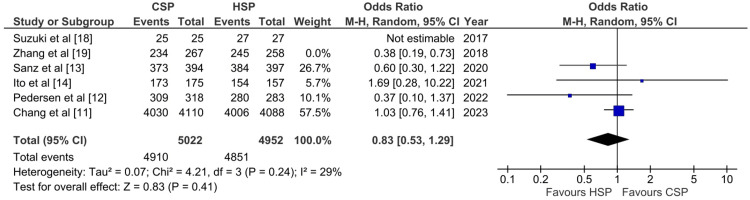
Forest plot of CSP versus HSP with the outcome of en bloc resection without the Zhang et al. study CSP: cold snare polypectomy; HSP: hot snare polypectomy

**Figure 11:**
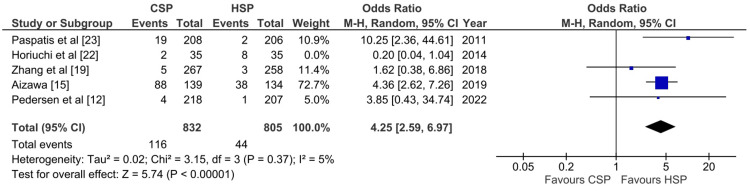
Forest plot of CSP versus HSP with the outcome of intraprocedural bleeding per patient without the Horiuchi et al. study CSP: cold snare polypectomy; HSP: hot snare polypectomy

## Appendices

Appendix 1

Appendix 2 
